# Revealing the astragalin mode of anticandidal action

**DOI:** 10.17179/excli2020-2987

**Published:** 2020-10-29

**Authors:** Marija Ivanov, Abhilash Kannan, Dejan Stojkovic, Jasmina Glamoclija, Simona Golic Grdadolnik, Dominique Sanglard, Marina Sokovic

**Affiliations:** 1 Department of Plant Physiology, Institute for Biological Research "Siniša Stankovic"- National Institute of Republic of Serbia, University of Belgrade, Bulevar Despota Stefana 142, 11000 Belgrade, Serbia; 2 Institute of Microbiology, University Hospital Lausanne and University Hospital Center, Rue du Bugnon 48, Lausanne, Switzerland; 3 Laboratory for Molecular Structural Dynamics, National Institute of Chemistry, Hajdrihova ulica 19, 1000 Ljubljana, Slovenia

**Keywords:** astragalin, mode of action, antimicrobial, ergosterol biosynthesis, hyphal transition, membrane integrity

## Abstract

Due to limited arsenal of systemically available antifungal agents, infections caused by *Candida albicans* are difficult to treat and the emergence of drug-resistant strains present a major challenge to the clinicians worldwide. Hence further exploration of potential novel and effective antifungal drugs is required. In this study we have explored the potential of a flavonoid, astragalin, in controlling the growth of *C. albicans,* in both planktonic and biofilm forms by microdilution method; and in regulating the morphological switch between yeast and hyphal growth. Astragalin ability to interfere with membrane integrity, ergosterol synthesis and its role in the regulation of genes encoding for efflux pumps has been addressed. In our study, astragalin treatment produced good antimicrobial and significant antibiofilm activity. Anticandidal activity of astragalin was not related to *ERG11* downregulation, neither to direct binding to CYP51 enzyme nor was linked to membrane ergosterol assembly. Instead, astragalin treatment resulted in reduced expression of *CDR1* and also affected cell membrane integrity without causing cytotoxic effect on human gingival fibroblast cells. Considering that astragalin-mediated decreased expression of efflux pumps increases the concentration of antifungal drug inside the fungal cells, a combinatorial treatment with this agent could be explored as a novel therapeutic option for candidiasis.

## Abbreviations

ROS, reactive oxygen species; ABC, ATP-binding cassette; SDA/SDB, Sabouraud Dextrose Agar/Broth; MIC, Minimum inhibitory concentration; MFC, Minimum fungicidal concentration; CaCYP51, *Candida albicans* sterol 14α-demethylase; NBT, nitro blue tetrazolium; HGF, human gingival fibroblasts cells; PBS, Phosphate buffered saline; IC_50_, 50 % of cell viability when compared with untreated control; AmB, amphotericin B; qPCR, Quantitative Real-Time Polymerase Chain Reaction; FC, fold change.

## Introduction

There have been increasing incidences of invasive infections caused by *Candida spp* over the past few years with *C. albicans* being the most dominant pathogen. Invasive bloodstream infections caused by *Candida,* also known as candidemia, can result in mortality rates up to 54 % (Xiao et al., 2019[29]). Invasive nature of infection is driven by *C. albicans* ability to form biofilms - structures with tightly packed group of cells that remain resistant to antifungal treatments (Nett and Andes, 2020[14]). Upon attachment to the host cells, a characteristic feature of *C. albicans* is the ability to switch from avirulent yeast form of growth to invasive hyphal form. This pathogenic transition is accompanied by increased levels of reactive oxygen species (ROS) and expression of several hyphal specific genes and proteins (Schröter et al., 2000[19]; Thomas et al., 2020[28]). Several drugs are currently in use to combat candidiasis, but *Candida* *spp* are able to modify their response to antifungals to be able to survive the treatment. Common adaptation mechanisms of *C. albicans* in response to antifungals include **i)** increased expression of *CDR1* and *CDR2*, genes encoding multidrug efflux transporters of the ATP-binding cassette (ABC) transporter family, **ii)** upregulation of *MDR1*, a major facilitator transporter gene and **iii)** induction of ergosterol pathway through enhanced *ERG11/ CYP51* expression which is the main target of azoles (Sanglard et al., 2009[17]). All of the above mentioned adaptations by *C. albicans* present the requirement of higher doses of antifungal drugs for effective control (Kofla et al., 2011[10]). Additionally, high drug concentrations could also result in the development of major side effects among the treated patients (Scorzoni et al., 2017[21]), thereby making it important to find alternative ways to combat candidiasis. Considering the adaptational flexibilities of *C. albicans* towards antifungal treatment, there is an ever increasing need to search for novel compounds with antifungal properties. Compounds of both natural and synthetic origin have been examined thus far, but for majority of them a mode of action has not yet been revealed (Smiljkovic et al., 2019[23]). Flavonoids are a group of natural compounds found in various plants, teas, wines etc. and have wide range of biological properties, one of them being anticandidal activity (Smiljkovic et al., 2017[24]). Astragalin is a 3-O glucoside of kaempferol and can be found in different plants belonging to *Convolvulaceae*, *Ebenaceae*, *Rosaceae* and *Eucommiaceae* families. This compound is known to have anti-cancerous, cardioprotective and antioxidative properties (Riaz et al., 2018[16]) while its aglycone kaempferol has been studied for its positive anticandidal activities (Shao et al., 2016[22]). In this study, we investigate the role of astragalin as a novel anticandidal agent and uncover its mode of action towards the inhibition of *C. albicans* virulence factors.

## Material and Methods

### Microbial culture conditions

*C. albicans* was isolated from oral cavities of patients at the Clinic of Otorhinolaryngology, Clinical Hospital Centre Zvezdara, Belgrade, Serbia. Reference strain *C. albicans* ATCC 10231 was purchased from American Type Culture Collection. Strains were determined on CHROMagar plates (Biomerieux, France), grown on Sabouraud Dextrose Agar/Broth (SDA/SDB) (Merck, Germany) and deposited at the Mycological Laboratory, Department of Plant Physiology, Institute for Biological Research ''Siniša Stanković'', University of Belgrade.

### Anticandidal activity

Minimum inhibitory (MIC) and minimum fungicidal concentrations (MFC) of the compounds were determined by the modified microdilution technique (EUCAST, 2002[5]) in 96-well microtiter plates. Briefly, yeast cultures were diluted in sterile saline to a concentration of approximately 1.0 x 10^5^ CFU/per well. To determine the MIC and MFC's, microplates containing fungal cells in SDB medium were incubated with serial dilutions of compounds at 37 °C for 24 h. The MIC values represented the lowest concentrations of the compound at which no microscopic growth was observed. After serial sub-cultivations of 10 µl into microtiter plates containing 100 µl of SDB broth/well, and incubation at 37 °C for 24 h, MFC values were determined as the lowest concentrations with no visible growth, indicating 99.5 % killing of the original inoculum. Ketoconazole (Sigma Aldrich, Germany) was used as a positive control. Flavonoid compound astragalin (kaemferol-3O-glucoside) was commercially bought (Extrasynthese, France). 

Since the tested strains exhibited similar sensitivity to anticandidal treatment strain *C. albicans* 475/15 was selected as reference strain for all the further experiments. 

### Antibiofilm activity 

Impact of astragalin on biofilm formation was determined as described by Cady et al. (2012[3]) with some modifications. *C. albicans* 475/15 cells were incubated at MIC and subMIC concentrations of the compounds in 96 well microtiter plates with adhesive bottom (Sarstedt, Germany) at 37 ºC. After 24 hours of incubation wells were washed twice with sterile PBS (Phosphate buffered saline, pH 7.4) and cells were fixed with methanol. After fixation methanol was discarded, plate was air dried and stained with 0.1 % crystal violet (Bio-Merieux, France). After 30 min plate was washed with water to remove any unbound stain and air dried. Ethanol 96 % (Zorka, Serbia) was added to dissolve the bound stain. Absorbance was read on Multiskan™ FC Microplate Photometer, Thermo Scientific™ and the percentage of inhibition of biofilm formation was calculated by the formula specified below: 

[(A_620_control- A_620_sample) / A_620_control] × 100.

### Measurement of membrane permeability 

The impact of astragalin on membrane permeability of *C. albicans* 475/15 was determined according to Tang et al. 2008[27], with some modifications. *C. albicans* 475/15 was incubated overnight at 37 °C, washed and suspended in 10 mM PBS (pH 7.4). Density of cells was adapted to 10^8^ CFU/mL. *C. albicans* was incubated with astragalin at 1½ MICs for: 0, 15, 30, 45 and 60 min; astragalin dissolved in PBS was used as blank. The mixture was filtered through 0.22 μm pore size filter and optical density was recorded at 260 nm and 280 nm with Agilent/HP 8453 UV-Visible Spectrophotometer Agilent Technologies, USA, at room temperature.

### Ergosterol binding as potential mode of action

Microdilutions of astragalin and amphotericin B (AmB, positive control) were prepared in the same manner as used for determination of antimicrobial activity, except that ergosterol (400 µg/mL) was added to the rows of the plate (Leite et al., 2015[11]). MIC values were observed after incubation at 37 ºC for 24 h, and were compared with the MIC value of samples without ergosterol addition. 

### Binding affinities of astragalin towards CaCYP51 enzyme

The enzyme sterol 14α-demethylase (CYP51) was previously isolated from *C. albicans* (CaCYP51) and kindly provided by Laboratory for Molecular Structural Dynamics, National Institute of Chemistry, Ljubljana, Slovenia. Binding properties of astragalin and positive control, ketoconazole (0, 2, 8, 16, 32, 64, 128, 256, 300 µM) were investigated using UV-Visible spectroscopy. Compounds were mixed with CaCYP51 protein and spectra were recorded in 350-500 nm range with potential ligand-induced spectral changes observed as difference type II spectral responses (Zelenko et al., 2014[30]). 

### RNA isolation and reverse transcription 

Total RNA was extracted from 5 mL logarithmic-phase cultures grown in YEPD medium, using RNeasy Protect Mini kit (Qiagen) by a process involving mechanical disruption of the cells with glass beads as previously described (Sanglard et al., 1999[18]). The concentration and purity of the RNA was determined using a UV spectrophotometer (NanoDrop; ThermoFisher Scientific) by measuring the absorbance at 230 (OD_230_), 260 (OD_260_) and 280 nm (OD_280_). The OD_260nm_/OD_280nm_ of the samples, reflecting the average purity, ranged from 1.80 to 2.05, and the OD_260nm_/ OD_230nm_ was in the range of 2.00-2.60. For qPCR, 1 µg RNA was reverse transcribed to cDNA using the Transcriptor High Fidelity cDNA synthesis kit (Roche) involving random hexamer as a priming method. Prior to reverse transcription reaction, the total RNA samples were treated with DNase from DNA-free^TM^ DNA removal kit for 30 min at 37 °C (Invitrogen) according to the manufacturer's instructions. 

### qPCR

qPCR reactions were performed with 0.2 µM of each primer and 0.2 µM of probe for genes *ACT1*, *CDR1*, *MDR1* and *ERG11* (see Supplementary material), and the iTAQ Supermix with ROX (BioRad, Reinach, Switzerland) according to the manufacturer's instructions using StepOnePlus^TM ^Real Time PCR System (Life Technologies). The expression level of *ACT1* was used for normalization, and fold change (FC) values were calculated for *CDR1*, *MDR1* and *ERG11* *in vitro* in the absence and presence of the compounds in their previously determined minimal inhibitory concentrations for 30 min. Technical triplicates were included in each reaction and all the reactions were repeated twice on biological replicates.

### Determination of intracellular ROS levels in C. albicans 475/14 

The impact of astragalin on levels of intracellular ROS was determined according to the method described by Paez et al. (2010[15]). *C. albicans* 475/15 was incubated with MIC of astragalin overnight at 37 ºC; 0.5 mL of 1 mg/mL nitro blue tetrazolium (NBT) was added and incubation was continued at 37 ºC for 30 min. After addition of 0.1 mL 0.1 M HCl tubes were centrifuged at 2500 g for 10 min. Dimethyl sulfoxide (0.6 mL) and phosphate saline buffer (0.8 mL) was added to the pellet and absorbance was recorded at 575 nm using Agilent/HP 8453 UV-Visible Spectrophotometer (Agilent Technologies, USA).

### Potential of astragalin to interfere with C. albicans yeast to hyphal transition

*C. albicans* 475/15 cells were incubated with MIC of astragalin in YPD + 10 % FBS. After 4 h of incubation at 37 ºC, cells were observed under microscope (Nikon Eclipse TS2, Netherlands) and number of cells growing in the yeast or hyphal and germ tube form was determined and presented as percentage of hyphal cells. Assay was performed in triplicate.

### Cytotoxicity assay

Crystal violet assay was used for the determination of cytotoxic effect, according to the previous protocol (Feoktistova et al., 2016[8]), with slight modifications. We used human gingival fibroblast cells HGF-1 (ATCC® CRL-2014™) for the assay. Cells 4 × 10^3^/well were seeded in a 96-well plate and incubated for 48 h at 37˚C to enable adhesion of cells to the wells. After, the medium was removed from the wells, and 100 µL/well of fresh medium supplemented with different concentrations of astragalin (0.0125 - 0.4 mg/ well) was added to the cells. The cells were treated in triplicate wells for each condition and further incubated for 24 h. The medium was removed and the cells were washed twice with PBS. Then, 100 µL of 0.4 % crystal violet staining solution was added to each well, and incubated for 20 min at room temperature. Crystal violet was removed, the plates were washed in a stream of tap water and left to dry. 100 µL of methanol was added to each well in order to dissolve the dye. Optical density of each well was measured at 570 nm (OD570) with a plate reader. The results were expressed as IC_50_ value, indicating 50 % of cell viability when compared with untreated control. The solvent was used as a negative control. The criterion used to categorize the cytotoxicity to HGF-1 cells was as follows: IC_50_ ≤ 20 µg/mL = highly cytotoxic, IC_50_ ranged between 31 and 200 µg/mL = moderately cytotoxic, IC_50_ ranged between 201 and 400 µg/mL = weakly cytotoxic, and IC_50_ > 401 µg/mL = no cytotoxicity.

## Results and Discussion

### Promising antifungal and antibiofilm activity of astragalin

MIC and MFC values of astragalin and ketoconazole were determined by serial dilutions and incubation for 24 h. We observed that astragalin could inhibit fungal growth. Majority of the strains were identically susceptible to astragalin at MIC value of 0.075 mg/mL, while *C. albicans* ATCC 10231 had MIC 0.125 mg/mL (Table 1(Tab. 1)). The average MFC values for all strains were twice as high as the MIC values (Table 1(Tab. 1)). In previous studies, ethyl acetate extract of *Helichrysum compactum* was shown to have promising anticandidal activity (MIC 0.04 mg/mL) which could be linked to the high concentration of astragalin observed in this extract (28 mg/kg) (Süzgeç et al., 2005[25]). Likewise, astragalin extracted from *Bellis sylvestris* leaves was also able to inhibit more than 20 % growth of *C. albicans* ATCC 10231 at a concentration of 0.128 mg/mL (Scognamiglio et al., 2016[20]). Surprisingly, some of the earlier studies had reported the lack of antimicrobial activity of astragalin towards ATCC strains 10231 (Süzgeç-Selçuk and Birteksöz et al., 2011[26]) and 14053 even at concentrations as high as 0.5 mg/mL (Fattouch et al., 2007[7]). The differences in these findings could be attributed to diverse methodologies (described by authors) or high diversity among strains selected for testing (Süzgeç et al., 2005[25]; Scognamiglio et al., 2016[20]; Süzgeç-Selçuk and Birteksöz, 2011[26]; Fattouch et al., 2007[7]). 

**Figure 1:**
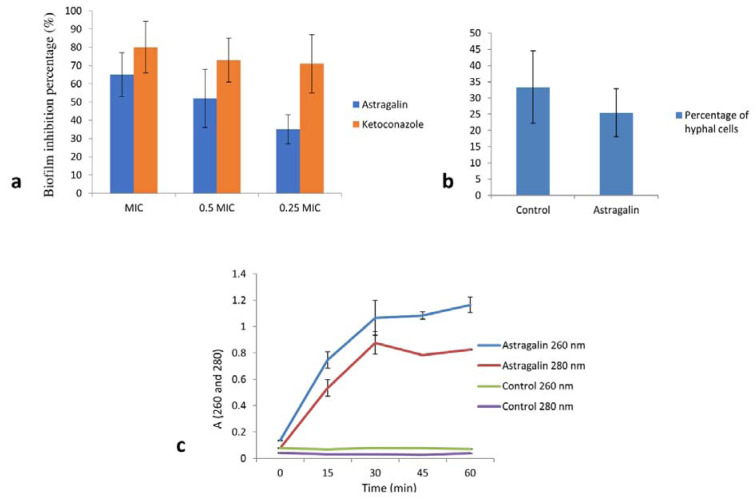
a) Inhibition of biofilm formation after treatment with astragalin and ketoconazole, expressed as inhibition percentage (100 % means no biofilm is established). b) Percentage of hyphal cells after 4 h treatment, control is *C. albicans* without any treatment. c) Nucleotide leakage detected by measuring the absorbance at wavelengths 260 nm and 280 nm after treatment of *C. albicans* cells with 1½ MIC of astragalin for 0, 15, 30, 45 and 60 min. All values represent means ± SD of three replicates.

We then assessed the antibiofilm activity of astragalin on strain *C. albicans* 475/15. In this regard, fungal cells were once again incubated in the absence and presence of astragalin at different concentrations (MIC, 0.5 MIC and 0.25 MIC). Astragalin induced a significant reduction in the ability of *C. albicans* to form biofilms (Figure 1a(Fig. 1)). The results revealed more than 50 % reduction in the biofilm biomass after incubation of the fungal cells with 0.5 MIC of astragalin, compared to the untreated biofilm and about 65 % reduction with doses equal to MIC (Figure 1a(Fig. 1)). Interestingly, one of previous studies showed no antibiofilm activity of astragalin (resulted in less than 10 % of biofilm inhibition) (Scognamiglio et al., 2016[20]). The effect of astragalin in prevention of biofilm formation found in this study could be explored further since inhibition of this pathogenic trait could lead to lower virulence of fungus. In conclusion, our findings clearly highlighted the antifungal potential of astragalin. 

### Impact of astragalin on yeast to hyphal transition

In our study, astragalin did not show any significant anti-oxidative activity in lowering the ROS production in *C. albicans*. We observed only 6 % reduction in ROS production compared to the 40 % inhibition in ROS activity of previously published flavonoids apigenin and apigetrin in an identical assay (Smiljkovic et al., 2017[24]). We also performed the microscopic examination on the growth of fungal cells in the presence and absence of astragalin and determined the number of cells in yeast and hyphal forms. The percentage of hyphal cells was marginally reduced following 4 h treatment with astragalin at MIC (Figure 1b(Fig. 1)). A previous study using honey flavonoid extract containing kaempferol had demonstrated its ability to reduce the number of cells switching from yeast to hyphal growth (Candiracci et al., 2012[4]).

### Assessing the binding of astragalin to ergosterol and its ability to interfere with sterol biosynthesis

To determine whether astragalin interacts with fungal ergosterol, its MIC against *C. albicans* 475/15 was determined both with and without the addition of exogenous ergosterol to the culture medium. We found that the MIC of AmB (used as a positive control) was three times higher when ergosterol was added, unlike MIC of astragalin which was not influenced by the presence of ergosterol (Table 2(Tab. 2)).

**Figure 2:**
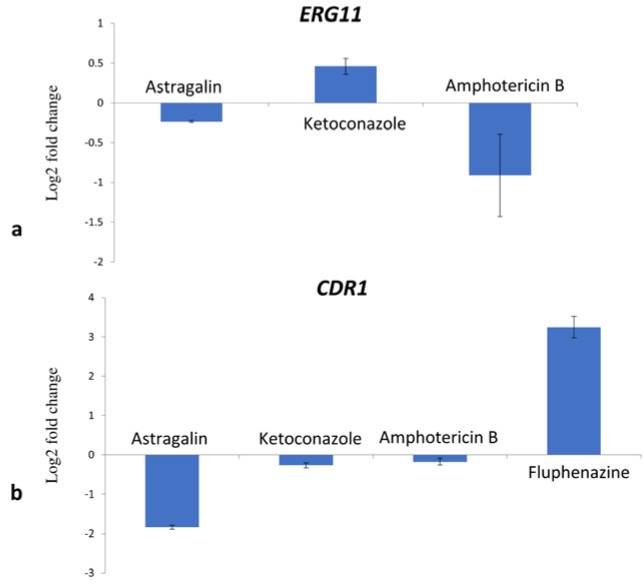
Expression levels of *ERG11* (a) and *CDR1* (b) after treatment with MIC of astragalin, ketoconazole and amphotericin B; fluphenazine was used as positive control for *CDR1* expression. Values are expressed as Log_2_fold change (log2 FC) of RQ values and presented as an average of two biological replicates.

These results suggest that astragalin did not directly interact with ergosterol and that the inhibition of fungal growth was likely caused by other mechanisms. To study the binding potential of astragalin to CYP51 differences in the spectral response was recorded before and after the addition of compound to CYP51 protein. We observed that astragalin did not show any binding affinity for fungal CYP51 (Table 2(Tab. 2)), once again suggesting that antifungal activity of astragalin was not mediated by its interference with the ergosterol biosynthesis. We then assessed the impact of astragalin on *ERG11* expression by Quantitative Real-Time Polymerase Chain Reaction (qPCR). Following 30 min treatment of the fungal cells with the compounds, astragalin treatment failed to produce any significant changes in *ERG11* mRNA levels (less than 0.5 log2 FC) (Figure 2a(Fig. 2)). Compared to the untreated cells, ketoconazole caused only a slight increase (0.5 log2 FC) in *ERG11* levels. We believe that longer treatment period (> 30 min) was probably necessary for stronger induction of the gene. AmB also reduced the expression of *ERG11*, albeit to a lesser extent (< 1 log2FC). These results suggest that besides binding to ergosterol, AmB reduced the ergosterol content in the cell to contribute to the antifungal activity. Based on the above observations, we believe that astragalin activity is not directly related to the inhibition of ergosterol biosynthesis.

### Impact of astragalin on expression levels of genes coding for efflux pumps 

*CDR1* and *MDR1* expression was tested at the mRNA level after induction of the strain *C. albicans* 475/15 with the compounds at their MIC concentrations for 30 min. Astragalin reduced *CDR1* levels in the fungal cells (> 1.5 log2 FC) **(**Figure 2b(Fig. 2)**)**. On the other hand, ketoconazole and AmB failed to produce any significant changes to *CDR1* expression, possibly since the induction of *CDR1* with azoles required longer incubation period to increase the expression of efflux pumps (Liu and Myers, 2017[12]) (Figure 2b(Fig. 2)). Likewise, changes in the expression levels of *MDR1* due to astragalin treatment could not be detected since the strain did not have any basal expression of this gene (data not shown). Astragalin was shown to be a potential inhibitor of P-glycoprotein, a protein involved in efflux of drugs (Ammar, 2017[1]). Additionally, other kaempferol derivatives such as kaempferol-3-O-β-d-(6″-E-p-coumaroyl) glucopyranoside and kaempferol 3-*O*-α-L-(2,4-bis-*E*-*p*-coumaroyl) rhamnoside, were shown to inhibit NorA efflux pump of *Staphylococcus aureus* (Falcão-Silva et al., 2009[6]; Holler et al., 2012[9]). The ability of kaempferol to inhibit bacterial efflux pumps was reported in one of the studies at an IC_50 _of 19 µg/mL (Brown et al., 2015[2]) which is lower than concentration of astragalin used in this study (75 µg/mL). The downregulation of *CDR1, CDR2 *and* MDR1 *by kaempferol has also been well documented (Shao et al., 2016[22]). Astragalin's ability to reduce the expression of *CDR1* is of potential interest when developing novel antifungal therapies. A compound that can reduce drug efflux and possess good antifungal effect could be a promising strategy towards candidiasis treatment.

### Effect of astragalin on cell membrane integrity

We studied the nucleotide leakage as a measure of astragalin impact on fungal cell membrane integrity by treating the fungal cells with astragalin for varying amounts of time. We observed strong absorbance signals for nucleic acids (A_260_) and proteins (A_280_) soon after 30 min treatment (Figure 1c(Fig. 1)). This clearly highlighted a strong effect of astragalin in reducing the fungal membrane integrity, thereby contributing to its antifungal activity. Similarly, astragalin was shown to cause morphological changes to the cell membrane in the parasite *Trypanosoma cruzi* (Marín et al., 2011[13]). Surprisingly, our finding was not in agreement with an earlier *in silico* study that predicted astragalin to function as membrane integrity agonist (Ammar, 2017[1]) indicating that *in silico* docking studies should be followed by at least some *in vitro* evaluations before predicting biological activities of different compounds.

### Astragalin is not cytotoxic to human fibroblast cells

Cytotoxic activity of the compound was tested on HGF-1 cells (Table 2(Tab. 2)). Astragalin did not exhibit any cytotoxicity up to 400 μg/mL. The fact that inhibition of fungal growth is achieved with astragalin in concentration of 75 μg/mL (Table 2(Tab. 2)) makes this flavonoid a promising candidate as safe antifungal compound for potential use in human medicine.

## Conclusions

Astragalin, one of the flavonoids found in various plants, was proven as an anticandidal agent. Our results clearly demonstrated promising antimicrobial activities of astragalin and its ability to reduce fungal biofilm formation, which is one important factor promoting *Candida* virulence, without significantly impacting the yeast to hyphae transition. Furthermore, we explored the astragalin's anticandidal mode of action in more details. Our finding suggested that astragalin acted against the fungal plasma membrane by interfering with the membrane integrity and was able to downregulate the expression of *CDR1*. These mechanisms could be further investigated *in vitro* and *in vivo* in order to classify and develop compound as a therapeutic option. In this perspective, the lack of cytotoxic activity of astragalin on human fibroblasts is of potential interest. Our results confirm that astragalin could be used in combination with other known inhibitors of efflux pumps and commercial antifungals to provide significant antimicrobial effect at lower drug concentrations in the effective treatment of *C. albicans* infections.

## Declaration of interest

The authors report no conflicts of interest. The authors alone are responsible for the content and writing of this article.

## Acknowledgement

This work is supported by the Serbian Ministry of Education, Science and Technological Development for financial support (Grant number 451-03-68/2020-14/200007). Binding study to CaCYP51 was supported by the Slovenian Research Agency (Grant numbers J1-8145 and P1-0010) and by a program of scientific and technological cooperation between the Republic of Serbia and the Republic of Slovenia “A combined methodology towards the development of novel, selective inhibitors of Candida CYP51”. The authors are grateful to the FEMS for providing FEMS Research and Training Grant (FEMS-GO-2017-015) to Mrs Marija Ivanov for her visit to the Institute of Microbiology, University Hospital Lausanne and University Hospital Center, Rue du Bugnon 48, Lausanne, Switzerland.

## Supplementary Material

Supplementary material

## Figures and Tables

**Table 1:**
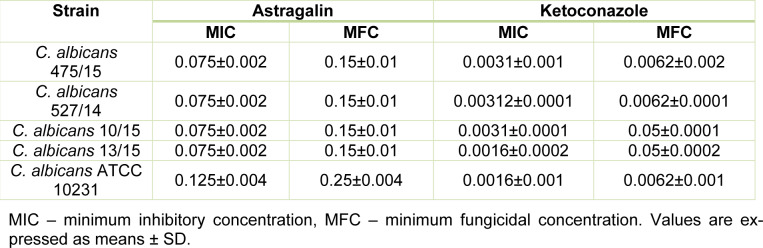
Antimicrobial activity of compounds (results represent the MIC and MFC values in mg/mL)

**Table 2:**
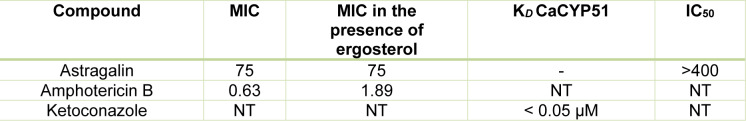
Minimal inhibitory concentrations (µg/mL) of astragalin and amphotericin B towards *C. albicans* in the absence and presence of ergosterol; dissociation constants (K*_D_*) after titration of *C. albicans* CYP51 (CaCYP51) with tested compounds; cytotoxicity of astragalin towards human gingival fibroblasts (IC_50_ μg/mL). NT - not tested

## References

[1] Ammar O (2017). In silico pharmacodynamics, toxicity profile and biological activities of the Saharan medicinal plant Limoniastrum feei. Braz J Pharm Sci.

[2] Brown AR, Ettefagh KA, Todd D, Cole PS, Egan JM, Foil DH (2015). A mass spectrometry-based assay for improved quantitative measurements of efflux pump inhibition. PLoS One.

[3] Cady NC, McKean KA, Behnke J, Kubec R, Mosier AP, Kasper SH (2012). Inhibition of biofilm formation, quorum sensing and infection in Pseudomonas aeruginosa by natural products-inspired organosulfur compounds. PLoS One.

[4] Candiracci M, Citterio B, Piatti E (2012). Antifungal activity of the honey flavonoid extract against Candida albicans. Food Chem.

[5] EUCAST (European Committee on Antibiotic Susceptibility) (2002). Method for determination of minimal inhibitory concentration (MIC) by broth dilution of fermentative yeasts. Discussion document E. Dis. 7.1.

[6] Falcão-Silva VS, Silva DA, Souza Mde F, Siqueira-Junior JP (2009). Modulation of drug resistance in Staphylococcus aureus by a kaempferol glycoside from Herissantia tiubae (Malvaceae). Phytother Res.

[7] Fattouch S, Caboni P, Coroneo V, Tuberoso CI, Angioni A, Dessi S (2007). Antimicrobial activity of Tunisian quince (Cydonia oblonga Miller) pulp and peel polyphenolic extracts. J Agr Food Chem.

[8] Feoktistova M, Geserick P, Leverkus M (2016). Crystal violet assay for determining viability of cultured cells. Cold Spring Harb Protoc.

[9] Holler JG, Christensen SB, Slotved HC, Rasmussen HB, Gúzman A, Olsen CE (2012). Novel inhibitory activity of the Staphylococcus aureus NorA efflux pump by a kaempferol rhamnoside isolated from Persea lingue Nees. J Antimicrob Chemoth.

[10] Kofla G, Turner V, Schulz B, Storch U, Froelich D, Rognon B (2011). Doxorubicin induces drug efflux pumps in Candida albicans. Med Mycol.

[11] Leite MC, de Brito Bezerra AP, de Sousa JP, de Oliveira Lima E (2015). Investigating the antifungal activity and mechanism(s) of geraniol against Candida albicans strains. Med Mycol.

[12] Liu Z, Myers LC (2017). Mediator tail module is required for Tac1-activated CDR1 expression and azole resistance in Candida albicans. Antimicrob Agents Ch.

[13] Marín C, Ramírez-Macías I, López-Céspedes A, Olmo F, Villegas N, Díaz JG (2011). In vitro and in vivo trypanocidal activity of flavonoids from Delphinium staphisagria against Chagas disease. J Nat Prod.

[14] Nett JE, Andes DR (2020). Contributions of the biofilm matrix to Candida pathogenesis. J Fungi.

[15] Paez PL, Becerra MC, Albesa I (2010). Effect of the association of reduced glutathione and ciprofloxacin on the antimicrobial activity in Staphylococcus aureus. FEMS Microbiol Lett.

[16] Riaz A, Rasul A, Hussain G, Zahoor MK, Jabeen F, Subhani Z (2018). Astragalin: a bioactive phytochemical with potential therapeutic activities. Adv Pharmacol Sci.

[17] Sanglard D, Coste A, Ferrari S (2009). Antifungal drug resistance mechanisms in fungal pathogens from the perspective of transcriptional gene regulation. FEMS Yeast Res.

[18] Sanglard D, Ischer F, Calabrese D, Majcherczyk PA, Bille J (1999). The ATP binding cassette transporter GeneCgCDR1 from Candida glabrata is involved in the resistance of clinical isolates to azole antifungal agents. Antimicrob Agents Ch.

[19] Schröter C, Hipler UC, Wilmer A, Künkel W, Wollina U (2000). Generation of reactive oxygen species by Candida albicans in relation to morphogenesis. Arch Dermatol Res.

[20] Scognamiglio M, Buommino E, Coretti L, Graziani V, Russo R, Caputo P (2016). Phytochemical investigation and antimicrobial assessment of Bellis sylvestris leaves. Phytochem Lett.

[21] Scorzoni L, de Paula E, Silva AC, Marcos CM, Assato PA, de Melo WC (2017). Antifungal therapy: new advances in the understanding and treatment of mycosis. Front Microbiol.

[22] Shao J, Zhang M, Wang T, Li Y, Wang C (2016). The roles of CDR1, CDR2, and MDR1 in kaempferol-induced suppression with fluconazole-resistant Candida albicans. Pharm Biol.

[23] Smiljkovic M, Kostic M, Stojkovic D, Glamoclija J, Sokovic, M (2019). Could flavonoids compete with synthetic azoles in diminishing Candida albicans infections? A comparative review based on in vitro studies. Curr Med Chem.

[24] Smiljkovic M, Stanisavljevic D, Stojkovic D, Petrovic I, Marjanovic Vicentic J, Popovic J (2017). Apigenin-7-O-glucoside versus apigenin: Insight into the modes of anticandidal and cytotoxic actions. EXCLI J.

[25] Süzgeç S, Meriçli AH, Houghton PJ, Cubukçu B (2005). Flavonoids of Helichrysum compactum and their antioxidant and antibacterial activity. Fitoterapia.

[26] Süzgeç-Selçuk S, Birteksöz AS (2011). Flavonoids of Helichrysum chasmolycicum and its antioxidant and antimicrobial activities. S Afr J Bot.

[27] Tang YL, Shi YH, Zhao W, Hao G, Le GW (2008). Insertion mode of a novel anionic antimicrobial peptide MDpep5 (Val-Glu-Ser-Trp-Val) from Chinese traditional edible larvae of housefly and its effect on surface potential of bacterial membrane. J Pharmaceut Biomed.

[28] Thomas G, Bain JM, Budge S, Brown AJP, Ames RM (2020). Identifying Candida albicans gene networks involved in pathogenicity. Front Genet.

[29] Xiao Z, Wang Q, Zhu F, An Y (2019). Epidemiology, species distribution, antifungal susceptibility and mortality risk factors of candidemia among critically ill patients: a retrospective study from 2011 to 2017 in a teaching hospital in China. Antimicrob Resist Infect Control.

[30] Zelenko U, Hodošček M, Rozman D, Golič Grdadolnik S (2014). Structural insight into the unique binding properties of pyridylethanol(phenylethyl)amine inhibitor in human CYP51. J Chem Inf Model.

