# Supplementary material for: Revealing the astragalin mode of anticandidal action
Source: EXCLI J. 2020 Oct 29;19:1436–45. doi: 10.17179/excli2020-2987 (PMC7726490; doi:10.17179/excli2020-2987)
Supplement: Supplementary material [file EXCLI-19-1436-s-001.pdf]

EXCLI Journal 2020;19:1436-1445 – ISSN 1611-2156
Received: October 05, 2020, accepted: October 13, 2020, published: October 29, 2020

Supplementary material to:

REVEALING THE ASTRAGALIN MODE OF ANTICANDIDAL ACTION

Marija Ivanov[a,b], Abhilash Kannan[b], Dejan Stojković[a], Jasmina Glamočlija[a], Simona Golič Grdadolnik[c], Dominique Sanglard[b], Marina Soković[a*]

[a] Department of Plant Physiology, Institute for Biological Research "Siniša Stanković"- National Institute of Republic of Serbia, University of Belgrade, Bulevar Despota Stefana 142, 11000 Belgrade, Serbia
[b] Institute of Microbiology, University Hospital Lausanne and University Hospital Center, Rue du Bugnon 48, Lausanne, Switzerland
[c] Laboratory for Molecular Structural Dynamics, National Institute of Chemistry, Hajdrihova ulica 19, 1000 Ljubljana, Slovenia

* **Corresponding author:** Dr. Marina D. Soković, Full Research Professor, Department of Plant Physiology, Institute for Biological Research „Siniša Stanković"- National Institute of Republic of Serbia, University of Belgrade, Bulevar Despota Stefana 142, 11000 Belgrade, Serbia; Phone: +381 11 207 84 19; Fax: +381 11 2 761 433; E-mail: mris@ibiss.bg.ac.rs

http://dx.doi.org/10.17179/excli2020-2987

This is an Open Access article distributed under the terms of the Creative Commons Attribution License (http://creativecommons.org/licenses/by/4.0/).

Supplementary Table 1: Sequences of TaqMan primers and probes used in qPCR

Primer	Sequence
**CDR1-ORF-F**	ATGACTCGAGATATTTTGATA
**CDR1-ORF-R**	TTAACAGCAATGGTCTTTA
**MDR1-ORF-F**	GAGAAATATTTAGCCGATTAC
**MDR1-ORF-R**	TGTTATTGGAAGATGATGAT
**ERG11-ORF-F**	ATTGTTGAAACTGTCATTG
**ERG11-ORF-R**	CCCCTAATAATATACTGATCTG
**ACT-ORF-F**	GCATCACACTTTTTACAAT
**ACT-ORF-R**	AAACATAATTTGAGTCATCTTT
**Probe**	**Sequence**
**CDR1-P2**	CATTATGAGACCTGGTGAACTTACT
**MDR1-P2**	AACCAACTCTTGCTGATGATACAA
**ERG11-P2**	TTTGTCCCTTAGTGTTACACA
**ACT1-P2**	TTGCTCCAGAAGAACATCCAGT
